# *In situ* interrogation of microorganisms mediating hydrocarbon degradation

**DOI:** 10.1128/aem.02591-25

**Published:** 2026-02-25

**Authors:** Xin-Yue Ren, Jia-Heng Ji, Lingfei Hu, Peng Bao, Bin-Bin Xie, Shun Li, Niculina Musat, Florin Musat, Song-Can Chen

**Affiliations:** 1 State Key Laboratory of Soil Pollution Control and Safety, Zhejiang University 12377, Hangzhou, China; 2 MOE Key Laboratory of Environment Remediation and Ecological Health, College of Environmental and Resource Sciences, Zhejiang University 366101 , Hangzhou, China; 3 State Key Laboratory of Soil Pollution Control and Safety, College of Environmental and Resource Sciences, Zhejiang Provincial Key Laboratory of Agricultural Resources and Environment, Zhejiang University 366101 , Hangzhou, China; 4 State Key Laboratory of Regional and Urban Ecology, Institute of Urban Environment, Chinese Academy of Sciences 85406 , Xiamen, China; 5 University of Chinese Academy of Sciences 74519, Beijing, China; 6 State Key Laboratory of Bioreactor Engineering and School of Chemistry and Molecular Engineering, East China University of Science and Technology 47860, Shanghai, China; 7 Hainan Institute, Zhejiang University, Yazhou District, Sanya, China; 8 State Key Laboratory of Microbial Technology, Shandong University 520252, Qingdao, China; 9 Shandong Provincial Key Laboratory of Deep Sea Bioresources Exploration and Utilization, Shandong University 520252, Qingdao, China; 10 Department of Biology, Section for Microbiology, Aarhus University 683568, Aarhus, Denmark; The Pennsylvania State University, University Park, Pennsylvania, USA

**Keywords:** bioremediation, hydrocarbon degradation, microorganisms, functional guild, ecological network

## Abstract

Microbially mediated hydrocarbon biodegradation is a cornerstone of natural attenuation and engineered bioremediation, yet the *in situ* mechanisms and key microbial players remain incompletely resolved due to the historical reliance on cultivation-based approaches. Recent advances in cultivation-independent tools, particularly metagenomics, stable isotope probing (SIP), and single-cell techniques, now enable more effective identification of active microbial populations, their functional genes, and metabolic networks directly mediating hydrocarbon degradation *in situ*. These studies have unveiled a far greater phylogenetic and functional diversity than previously recognized, including the unexpected co-existence of alkane-oxidizing archaea and bacteria in similar environments. The underlying microbial actors exploit distinctive enzymes to initialize hydrocarbon oxidation under oxic and anoxic conditions and achieve complete degradation through complex ecological networks that involve cooperative and/or competitive interactions with other community members such as viruses. These findings offer better insights into the functioning of the microorganisms that control the fate of hydrocarbons *in situ* and, as a final outcome, help tailor bioremediation strategies for better performance.

## INTRODUCTION

Hydrocarbons are organic compounds composed exclusively of carbon and hydrogen, occurring in nature primarily as natural gas, crude oils, and coals. They are released into the biosphere via geological processes and from biological sources (methane gas) at a rate of over 230 Tg per year, primarily as gaseous alkanes and petroleum hydrocarbons (1, 2). However, anthropogenic discharges, driven by the rising demand of hydrocarbons for industrial and civil purposes, have been estimated to exceed the natural sources by an order of magnitude (2). The massive anthropogenic inputs have led to elevated atmospheric concentrations of gaseous alkanes (e.g., methane) and widespread contamination of marine and terrestrial ecosystems with petroleum hydrocarbons. Due to their chemical stability and recalcitrance, hydrocarbons often persist in environments for decades, imposing long-lasting threats on ecosystem biodiversity, service, and sustainability (3–5). These devastating impacts include disruption of global carbon cycle (6), exacerbation of climate change and tropospheric ozone formation (7), and habitat degradation through acute toxicity and bioaccumulation (8). Microorganisms capable of oxidizing hydrocarbons act as a critical natural sink of hydrocarbons, mitigating these effects through both aerobic and anaerobic degradation processes (9). By transforming hydrocarbons into carbon dioxide (CO_2_) and biomass, microorganisms could reduce the climate-forcing potential of methane and other gaseous alkanes and limit the transfer of toxic hydrocarbons through trophic chains. It is estimated that the microbially mediated hydrocarbon oxidation process removes ~90% of ~100 Tg subsurface-derived methane before it reaches the atmosphere and cleans up 50%–80% of ~758 million-liter oil released during major spill events (e.g., 2010 Deepwater Horizon incident) (10). Consequently, hydrocarbon-oxidizing microorganisms play a central role in the global carbon cycle, natural attenuation, and bioremediation and have been a major focus of environmental microbiology in the past decades.

**Fig 1:**
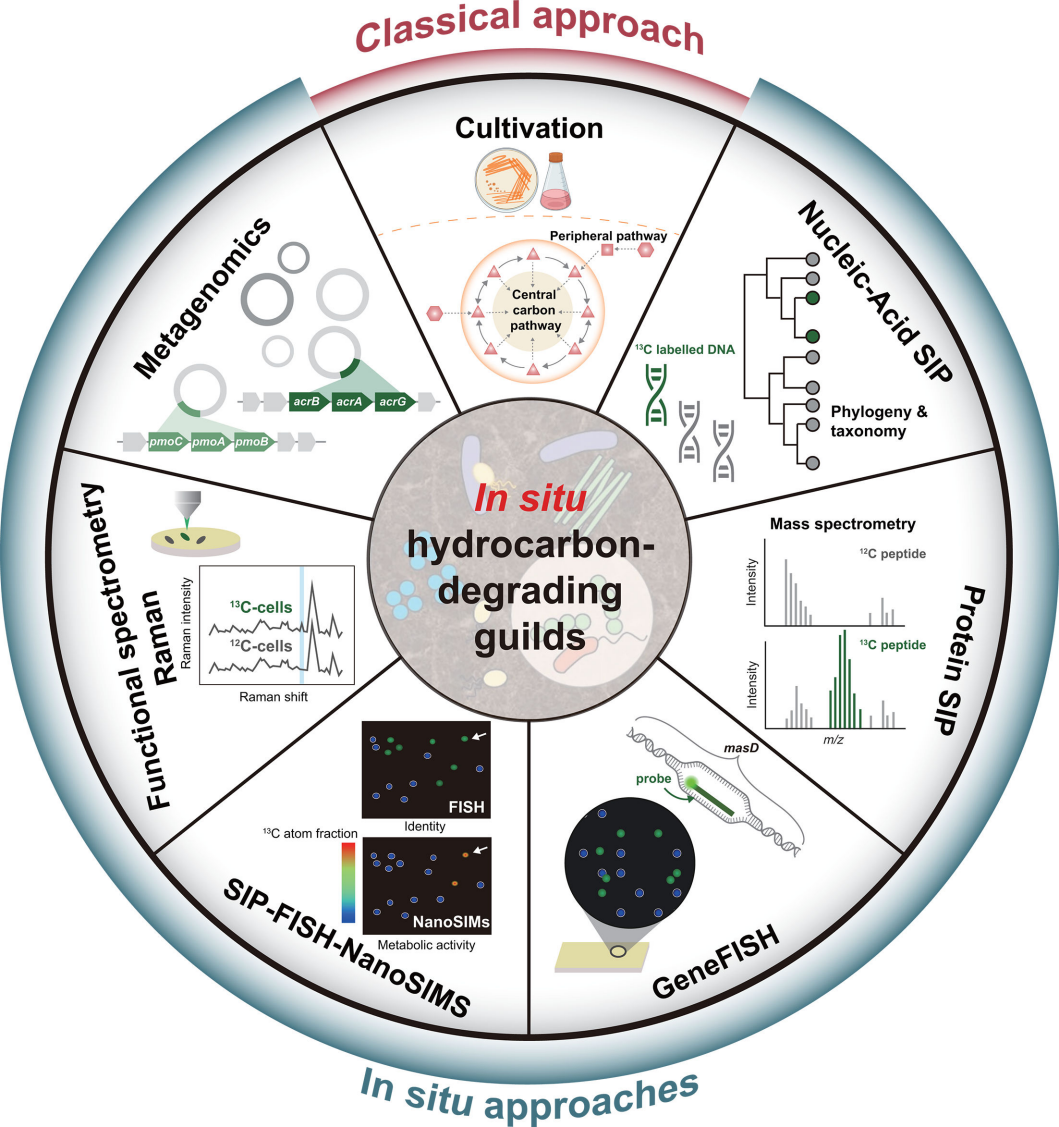
Overview of classical and *in situ* approaches for characterizing hydrocarbon-degrading functional guilds. Culture-independent techniques, grouped here as “*in situ* approaches,” include metagenomics, DNA-based stable isotope probing (DNA-SIP), protein-based stable isotope probing (protein-SIP), gene fluorescence *in situ* hybridization (gene-FISH), SIP combined with fluorescence *in situ* hybridization and nanoscale secondary ion mass spectrometry (SIP-FISH-NanoSIMS), and functional Raman spectroscopy. These methods permit researchers to capture the phylogenetic identity and genetic makeup of active hydrocarbon degraders within complex microbial communities. In contrast, cultivating techniques bring only a small fraction of the environmental microbiome into pure cultures, which might not represent the primary microorganisms responsible for hydrocarbon degradation *in situ*. Nevertheless, studies on pure cultures remain indispensable for the understanding of biochemical mechanisms underlying hydrocarbon oxidation.

Cultivation-dependent studies have acquired vast knowledge of the phylogeny, physiology, and biochemistry of hydrocarbon-degrading microorganisms (Fig. 1) (11, 12). Numerous phylogenetically distinct microorganisms, primarily from the *Pseudomonadota* (formerly *Proteobacteria*), *Actinomycetota* (formerly *Actinobacterota*), and *Bacillota* (formerly *Firmicutes*), have been isolated with the capability to degrade hydrocarbons under oxic or anoxic conditions (13). These microorganisms initialize the hydrocarbon degradation via various mechanisms, including oxygen-dependent hydroxylations by mono- or di-oxygenases or radical-based cleavage of C-H bonds followed by addition to fumarate, an oxygen-independent mechanism used by anaerobes (3, 5, 14–19). Genes encoding hydrocarbon activation and downstream degradation in those isolates have been extensively characterized and reviewed elsewhere (3, 20–23). Despite providing substantial progress in understanding the diversity and physiology of hydrocarbon degraders, cultivation-based approaches lack the capacity to link the identified microorganisms and their underlying metabolic pathways to biodegradation processes actually occurring *in situ*. Pure cultures functioning well in laboratory conditions often fail to predict the key players under real environmental conditions (11, 24, 25). Indeed, phylogenetic analyses of functional genes retrieved from soils via polymerase chain reaction (PCR) emphasized that uncharacterized microorganisms and divergent enzymes likely played an essential role in bioremediation (26). Moreover, cultivation-based approaches are not amenable to unraveling the multispecies degradation networks functioning at the community level, which control the flux of biodegradation reactions and determine the fate of hydrocarbons in the environments (27). Without information on the phylogenetic identity and specific genes or enzymes of metabolically active microbes in the context of ecological networks, natural attenuation and bioremediation deteriorate rapidly from a conceptually sound environmental technology into a highly empirical “black box” showing unpredictable outcomes (28).

During the past decades, a number of cultivation-independent techniques have been developed in microbial ecology, enabling researchers to track the ecologically relevant microorganisms in their native habitats. These include nucleic acid- or protein-based stable isotope probing (SIP) (29, 30), functional gene-targeted fluorescence *in situ* hybridization (gene-FISH) (31), FISH-nano scale secondary ion mass spectrometry (FISH-nanoSIMS) (32), biorthogonal non-canonical amino-acid tagging (BONCAT) (33), heavy water (D_2_O) integrated Raman microscopy (34), and emulsion paired isolation and concatenation PCR (epicPCR) (35). When coupled with metagenomics, these methods capture the genetic makeup of the active microbiome and unravel the metabolic interactions among community members *in situ* (36). In this review, we outline how these powerful techniques, in particular of SIP and metagenomics, are being applied to the field of hydrocarbon oxidation in environments. We summarize the main findings, including the newly discovered microorganisms, catabolic pathways, and complex degradation networks operating *in situ*. We anticipate that a deeper understanding of these processes offers new opportunities to tailor strategies of natural attenuation and bioremediation toward higher efficiency and reduced uncertainties.

## EMERGING TECHNIQUES TO TRACK THE HYDROCARBON OXIDIZERS IN COMPLEX MICROBIOMES

Metagenomics has emerged as a powerful tool to characterize hydrocarbon-degrading functional guilds, that is, assemblages of taxonomically diverse microorganisms that share common functional capacities in degrading hydrocarbons. Recent developments in sequencing techniques and computational algorithms allow the recovery of complete or near-complete genomes belonging to uncultured microorganisms directly from environments (37–40). Genome-based analyses offer an opportunity to pinpoint the hydrocarbon degradation potential among the vast majority of previously uncultivated microbial lineages and thereby provide a more comprehensive view of hydrocarbon-degrading populations in environments (41). To facilitate genome-centric analysis, Khot et al. (42) have developed the CANT-HYD database of marker genes (*n* = 37) involved in hydrocarbon degradation (42), which permits effective and accurate exploration of hydrocarbon degradation potential in genomes and metagenome-assembled genomes (MAGs). However, reconstructing microbial genomes from highly complex communities, such as those inhabiting soils, remains challenging due to the tremendous diversity and relatively uniform abundance distribution of genomes (43). To this end, metagenome data sets can also be analyzed in a gene-centric manner, where the hydrocarbon-degrading genes are identified and quantified within gene catalogs assembled from short reads. This strategy has revealed the relative abundance, taxonomic composition, and turnover of the functional guilds involved in hydrocarbon degradation within a specific biome (44) or along environmental gradients (e.g., salinity, pH, and oxygen) (45), while highlighting their role in sustaining a previously overlooked hydrocarbon cycling throughout the global ocean (46). A more targeted and cost-effective alternative to metagenomic sequencing is enabled by the epicPCR (35). It allows simultaneous amplification of the hydrocarbon-degrading genes and the taxonomic marker 16S rRNA genes from the same individual cell. Sequencing of the concatenated amplicons resolves the composition of functional guilds encoding hydrocarbon marker genes as well as their corresponding taxonomic affiliations. Exploration of sediments from marine cold seeps with epicPCR has unexpectedly linked the archaea-specific gene for anaerobic methane oxidation (*mcrA*) to bacteria (47). This suggests the existence of anaerobic bacterial methanotrophs, though this finding requires further experimental validation.

Metagenomics reveals the metabolic potential of microbiomes, yet it lacks the capacity to discern active populations from those dormant or inactive cells. SIP bridges this gap by tracing substrate assimilation into microbial biomass *in situ*. In hydrocarbon-focused SIP studies, the microbial communities are typically incubated with ^13^C-labeled hydrocarbons (e.g., ^13^C-alkanes), and the resulting heavy, isotope-enriched nucleic acids are separated by isopycnic centrifugation (48). Subsequent molecular profiling of the ^13^C-labeled fraction directly identifies the taxa actively driving substrate degradation. Early SIP studies relied on 16S rRNA gene amplicon sequencing of ^13^C-labeled DNA (49, 50), which only revealed the taxonomic identity of active hydrocarbon degraders. More recent advances coupled SIP with metagenomes, allowing reconstruction of the nearly complete genome and full catabolic pathways from the active degraders (51). However, DNA-based SIP often requires prolonged incubation (days to weeks) to achieve sufficient isotope incorporation, which greatly enhances the risks of cross-feeding, that is, the secondary labeling of non-target organisms via the uptake of labeled metabolic byproducts or dead biomass (52). In such cases, secondary consumers incorporate ^13^C from excreted metabolites or necromass of the primary degraders, leading to spurious labeling of non-primary degraders and overestimation of functional guild diversity. To mitigate these limitations, protein-SIP has emerged as a more rapid and physiologically informative alternative because isotopic incorporation into proteins occurs in a relatively shorter timeframe and the detection of ^13^C-labeled protein/peptide is more sensitive via mass spectrometry (48). All SIP variants remain constrained by high cost and commercial unavailability of ^13^C-labeled hydrocarbon substrates. To overcome these barriers, BONCAT has recently been adapted for activity-based profiling of hydrocarbon-degrading microbiomes. It exploited clickable, non-canonical amino acids, for example, L-homopropargylglycine (HPG), which are incorporated into proteins during active translation (53, 54). After brief incubation of environmental samples with HPG (with or without hydrocarbon substrates), translationally active cells conditioned on hydrocarbon addition are fluorescently labeled, sorted by fluorescence-activated cell sorting (FACS), and subjected to metagenomic sequencing. Application of BONCAT-FACS to coal-associated microbial communities has revealed distinctive hydrocarbon degradation pathways operating at high- and low-sulfate conditions (53).

Single-cell techniques now allow quantitative assessment of abundance and cell-specific catabolic activity of specific lineages within the complex microbial communities. Among these, geneFISH, a variant of FISH that employs polynucleotide probes targeting catabolic genes, has enabled direct enumeration of hydrocarbon-degrading guilds (55). For instance, geneFISH designed to target *masD*, which encodes the alkylsuccinate synthase involved in the anaerobic alkane oxidation pathway, identified and quantified alkane oxidizers in a microbial consortia (56). A complementary and more informative approach combines SIP with FISH-NanoSIMS (32). This approach provides taxonomic information on the same microbial cells through FISH, along with qualitative and quantitative assessments of their catabolic activities using NanoSIMS. A unique feature of the FISH-NanoSIMS approach is that it allows quantifying the flux of elements (e.g., carbon and nitrogen) among members of complex communities and thereby pinpointing quantitatively dominant players in substrate turnover. In hydrocarbon microbiology, the method has illuminated key alkane degraders in sulfate-reducing enrichment cultures (57), uncovered trophic interactions between archaea and bacteria in methane-oxidizing consortia (58, 59), and revealed putative syntrophic interactions between bacteria and archaea members in the ethane-oxidizing enrichment culture (60). More recently, FISH-NanoSIMS has been directly applied to marine sediments, quantifying the cell abundances and cell-specific hydrocarbon oxidation rate for three *Desulfosarcinaceae* clades capable of oxidizing short- and long-chain alkanes (61). Estimates based on these quantifications revealed these clades have the capacity to reduce nearly all sulfate not attributable to anaerobic methanotrophs. Further expanding the single-cell toolkits, Raman-activated cell sorting spectroscopy (RACS) has been integrated with SIP to discern the active functional microbes from complex communities in a non-destructive manner. The workflow labels the targeted functional guilds via SIP incubation, followed by sorting of isotopic labeled cells using Raman spectroscopy and reconstruction of the genome via single-cell sequencing. Application of this method identified active toluene degraders (*Pigmentiphaga*) and phenanthrene degraders (*Achromobacter* sp. and *Pseudomonas* sp.) in soils (62, 63). Moreover, the genome from Raman-SIP has guided the modification of the traditional cultivation medium, eventually bringing these non-canonical, yet active hydrocarbon degraders into pure culture (62, 63).

In summary, metagenomics bypasses the hurdles of cultivation, providing unprecedented access to the genetic potential of hydrocarbon degraders within microbiomes. While *in situ* activities can be further resolved through SIP and single-cell techniques, these activity-based approaches are often constrained by high costs, sample perturbation, and limited temporal resolution. An integration, therefore, is essential: utilizing high-throughput metagenomics for initial broad-scale exploration and narrowing down metabolic measurements to key targets. This combination maximizes the strengths of both approaches to provide a nuanced understanding of *in situ* hydrocarbon cycling.

## DIVERSITY PATTERNS OF HYDROCARBON-OXIDIZING FUNCTIONAL GUILDS IN THE ENVIRONMENT

### The uncultured majority and expanding phylogenetic diversity

Microorganisms capable of degrading hydrocarbons have been isolated from diverse environments, yet the ecological relevance of many cultured representatives and their contribution to *in situ* hydrocarbon cycling remain poorly understood. In fact, activity-based surveys in native habitats frequently uncover degraders that share limited taxonomic overlap with laboratory isolates. Notably, examples include (i) an uncultured *Alphaproteobacteria* clade UBA11222 actively driving the degradation of biphenyl in the PCB-contaminated river sediments (64); (ii) members from the class *Saccharimonadia* (formerly *Candidatus Saccharibacteri*a or TM7) associated with aerobic toluene degradation processes in soil environments (65, 66); and (iii) previously unrecognized *Alphaproteobacteria* and *Gammaproteobacteria* populations that proliferate in the polycyclic-aromatic-hydrocarbon (PAH)-degrading communities following the Deepwater Horizon oil spill (67). These uncultured microorganisms often dominate the active fraction of hydrocarbon-degrading functional guilds and exhibit high cell-specific hydrocarbon assimilation rates *in situ* (64). These observations highlight that a vast majority of ecologically relevant hydrocarbon degraders remain uncultured, implying a much higher diversity than currently represented by the cultivated representatives. Recent cultivation efforts complemented by extensive metagenomic studies have started to untap this hidden diversity, uncovering previously underappreciated roles, particularly of archaea, in both aerobic and anaerobic hydrocarbon degradation. These include (i) *Ca. Syntropharchaeum* from the class *Syntropharchaeia* capable of oxidizing propane and butane (68); (ii) *Ca. Alkanophaga* that can oxidize medium-chain petroleum n-alkanes (19); (iii) *Ca. Argoarchaeum* and *Ca. Ethanoperedens* within *Methanosarcinales* that perform ethane oxidation (60, 69); (iv) members of the class *Methanoliparia* that degrade long-chain alkanes, n-alkylcyclohexanes, and n-alkylbenzenes; (v) diverse uncultured members from *Methanomethylicia*, *Methanomassiliicoccales*, *Archaeoglobi*, *Bathyarchaeia*, *Helarchaeles,* and *Hadarchaeota*, all encoding genes for anaerobic hydrocarbon degradation (70–73); (vi) *Ca. Aerarchaeales* within the class *Syntropharchaeia* showing metabolic potential for aerobic hydrocarbon degradation (74); and (vii) *Ca. Alkanivorans nitratireducens*, an uncultured bacterium from the phylum *Bacillota* capable of oxidizing ethane, propane, and butane in wastewater treatment plants (75–77). Despite these exciting findings, the genome-inferred capacities of many newly discovered microbial lineages lack physiological or biochemical validation. For these novel clades, establishment of pure cultures or well-characterized enrichments remains indispensable for further metabolic characterization.

### Functional redundancy

**Fig 2:**
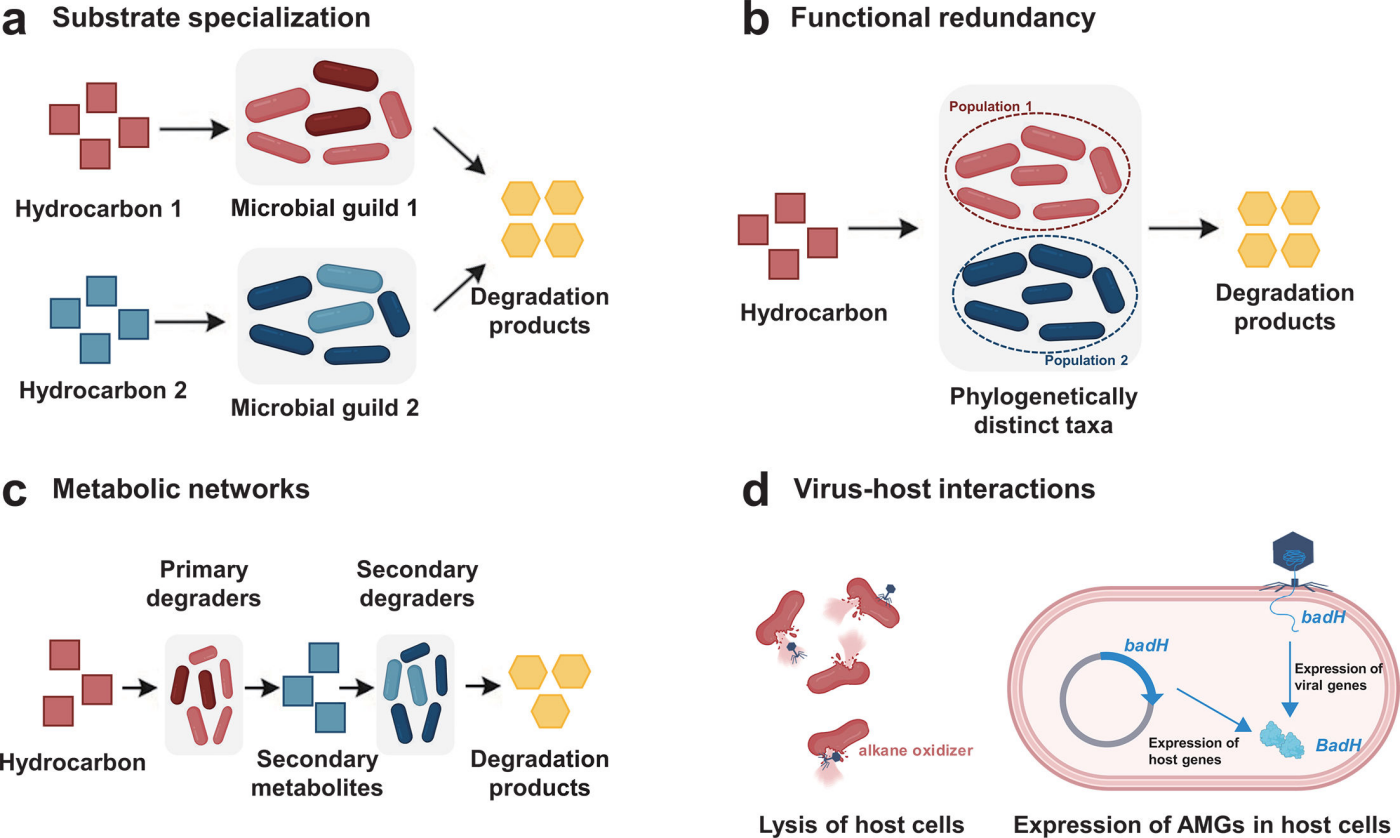
Ecology of *in situ* hydrocarbon degradation. (**a**) Individual components of the hydrocarbon mixture in soils are targeted by phylogenetically divergent hydrocarbon-degrading guilds; (**b**) many coexisting but taxonomically distinct microorganisms in environments encode the same hydrocarbon degradation functions; (**c**) hydrocarbon degraders form part of a complex ecological network, which involves direct and indirect metabolic interactions between community members; (**d**) viruses influence hydrocarbon cycling both directly through community population control viral lytic activities of host cells and indirectly through auxiliary metabolic genes (AMGs).

Hydrocarbon biodegradation in environments is concurrently performed by multiple phylogenetically distinct taxa (Fig. 2). A notable example is the co-occurrence of 75 bacterial genera in a contaminated pine root zone that are capable of deriving carbon from biphenyl (78). Although some of these genera might degrade biphenyl indirectly via growth on metabolic intermediates released by other community members (cross-feeding), convincing evidence indicated that more than one taxon functioned as a primary degrader. Indeed, co-existence of pollutant-degrading microorganisms was observed in nearly all SIP studies targeting specific functional types in soils, as well as in a wide range of ecosystems such as petroleum-associated deep-sea sediments (79–81) marine water (82), and wastewater (83). This functional redundancy may buffer the functioning of the ecosystem, allowing effective pollutant degradation to occur in a broad range of environmental conditions (84–86). For instance, toluene degradation in contaminated aquifers is maintained over broad temperature gradients by complementary *Bacillota* populations, with *Desulfosporosinus* dominating at low temperatures and the other members of *Bacillota* prevailing at high temperatures (87). Assuming different species within the functional group exhibit varying growth rates and efficiencies in metabolizing the contaminant hydrocarbons, shifts in memberships of functional groups following environmental perturbation may cause changes in the rate of contaminant biodegradation (88). Practical bioremediation strategies should aim to optimize the environmental conditions, for example, nutrient supply, that favor the dominance of functional microbes with highest metabolic activities toward targeted pollutants.

### Substrate specialization

Most contaminated environments contain complex mixtures of hydrocarbon compounds. Among these, individual components are often targeted by phylogenetically divergent hydrocarbon-degrading guilds (Fig. 2). For example, SIP experiments with multiple labeled substrates revealed that phenanthrene, anthracene, and fluoranthene in a forest soil were degraded by three distinct phylotypes, affiliated with *Sphingomonas*, *Rhodanobacter*, and *Acidobacteria*, respectively (89). In a bioreactor treating PAH-contaminated soil, low-molecular-mass PAHs (i.e., naphthalene and phenanthrene) were predominantly degraded by *Acidovorax*-, *Sphingobium*-, and *Pigmentiphaga*-related microorganisms, whereas Pyrene Group 2 (*Immundisolibacter*) was the principal microbial taxon involved in degradation of high-molecular-mass PAHs (i.e., pyrene and benz[a]anthracene) (90). This apparent partitioning of substrates to different community members suggests that shifts in the functional community structure should coincide with chemical changes of hydrocarbon residues during the biodegradation process in soils. Analogous ecological succession of oil-degrading bacteria driven by substrate specialization has been well documented following the Deepwater Horizon oil spill (91–93). During the initial stage of contamination, microbial communities in deep sea plumes were dominated by alkane-degrading bacteria of the genus *Bermanella* spp. and subsequently shifted to microbial populations capable of metabolizing more recalcitrant hydrocarbons (i.e., PAHs), such as *Cycloclasticus* and *Colwellia*. The substrate specialization nature of hydrocarbon-degrading bacterial communities suggests that bioremediation of complex hydrocarbon mixtures requires coordinated responses from diverse members.

### Functional community structure shaped by environmental conditions

Physio-chemical conditions in environments can strongly affect the structure of hydrocarbon-degrading microbial communities. Electron acceptor availability, substrate concentration, water depth, temperature, and salinity are among the key determinants (44, 94). Hydrocarbon-degrading microorganisms with distinct respiration metabolisms are selected based on the availability of electron acceptors (i.e., nitrate [NO₃⁻], ferric iron [Fe(III)], or sulfate [SO₄²⁻]). For example, sulfate-reducing bacteria, primarily of the genus *Desulfosporosinus*, were toluene degraders in an agricultural soil under sulfate-reducing conditions, whereas denitrifying bacteria within the family *Comamonadaceae* were the most abundant degraders under nitrate-reducing conditions (95). In wetland sediments, the addition of NO₃⁻ to sediment microcosms promoted the activity of anaerobic methanotrophs, whereas SO₄²⁻ and Fe(III) had minimal or inhibitory effects (96). Additionally, substrate concentration shapes the composition of the hydrocarbon-assimilating microbiome. In a coal-tar waste-contaminated sediment with low levels of benzene (10 p.p.m.), active degraders were identified as belonging to the class *Betaproteobacteria* (genera *Pelomonas* or *Ralstonia*). In contrast, with high benzene levels (200 p.p.m.), the dominant degraders belonged to *Gammaproteobacteria*, *Actinobacteria*, and *Alphaproteobacteria* (97). Beyond these abiotic factors, anthropogenic activities including historical oil spills and bioremediation practices significantly influence hydrocarbon-degrading microbial communities. Across the Baltic Sea subbasins, the composition of the hydrocarbon degradation genes was primarily shaped by oil spill history (44). Biostimulation treatments can directly reshape the composition of hydrocarbon-degrading populations *in situ*; for example, the addition of salicylate in a forest soil led to a transition of the dominant benzo[a]pyrene-metabolizing microbial populations from taxa affiliated with *Terrimonas* to those within the *Oxalobacteraceae* (98). Plant root exudates greatly modified the taxonomic composition of phenanthrene degraders in soil with dominance of *Sphingobium* in planted soil and *Sphingomonas* in bare soil (25, 99). These taxonomic variations in functional communities across a range of physiological conditions reflect niche differentiation of hydrocarbon-degrading microorganisms along axes other than hydrocarbon resources.

## *IN SITU* HYDROCARBON ACTIVATION MECHANISMS AND METABOLIC FEATURES OF INDIGENOUS DEGRADERS

### Hydrocarbon degradation pathways employed by the indigenous microbiomes

The uncultured majority of microbial communities harbor a variety of uncharacterized enzymes associated with hydrocarbon biodegradation. In particular, genes encoding novel ring hydroxylating dioxygenases (RHDs), which initiate the oxidation of various aromatic compounds (i.e., PAH or PCB), have been recovered from PAH-contaminated soils (100–102), tidal mudflats (103), and PCB-contaminated river sediments (78, 104) through metagenomics integrated with SIP. Most of these genes showed distant relatedness (<70% sequence identity) with those described in cultivated microorganisms. Cloning, heterologous overexpression, and function assays of RHD-encoding genes from soil environments demonstrated their competency toward oxidation of targeted hydrocarbons, which explained the observed biodegradation activities *in situ*. For example, a biphenyl RHD retrieved from the SIP-derived metagenomic library was shown to oxidize biphenyl and PCB congeners without chlorines at the 2 and 3 positions, giving insights into the metabolic potential of uncultured PCB/biphenyl degraders in polluted river sediments (104). Similarly, four PAH-RHDs retrieved from phenanthrene SIP experiments exhibited substrate preferences for two- and three-ring PAHs, including phenanthrene (102). Three of them hydroxylated phenanthrene on the C-1 and C-2 positions, rather than on C-3 and C-4 positions commonly observed among cultivated microorganisms, suggesting phenanthrene degradation may occur through alternative pathways in soils (102). These studies emphasize the so-far unrecognized catalytic properties of soil RHDs. Additionally, culture-based approaches, further combined with metagenomics, have also revealed that uncultured archaea employ a fundamentally distinct biochemical strategy for the anaerobic oxidation of alkanes compared to their bacterial counterparts. Hydrocarbon-degrading archaea activate alkanes using alkyl-coenzyme M reductase (ACR), an enzyme homologous to methyl-CoM reductase (MCR) (105). Targeted metabolite measurements have detected the presence of alkyl-coenzyme M in marine sediments incubated with hexadecane, supporting an active role of the archaea-mediated hydrocarbon degradation pathway in complex environments (106). However, progress in characterizing these alkane-activating enzymes remains limited, likely due to the lack of pure cultures and the difficulty of preserving their native biochemical properties.

### Unique metabolic features of indigenous hydrocarbon degraders

Microorganisms in the environment possess distinctive genomic features for hydrocarbon degradation. The catabolic genes obtained from metagenomics are generally found dispersed or shuffled compared to the typical operon structure observed in well-studied isolates. For instance, among a fosmid library constructed from an aged PAH-contaminated soil, nearly all clones contained novel organization of aromatic degradation genes, with various types of gene subsets that were dissimilar to pathway modules of known aromatic-utilizing bacteria (107). In parallel, genes for biphenyl dioxygenase subunits BphAE in a PCB-contaminated river sediment were found separated from other constituents of the *bph* operon (104). Segregation of catabolic genes into genomic regions suggested that degradation of biphenyl and hydrocarbon pollutants in the natural environment required concerted actions of various genomic fragmental pathways (108). These variations in genomic structure are probably the result of horizontal gene transfer and/or homologous recombination (109) and may have profound impacts on phenotypes, like the hydrocarbon-dependent growth characteristics of microorganisms highly relevant for bioremediation (110). Moreover, environmental genomics and physiological studies have revealed unique genomic and/or metabolic features of archaeal lineages that perform anaerobic oxidation of alkanes. Comparative genomics shows that anaerobic methanotrophic (ANME) archaea possess large multiheme cytochromes and specialized bioenergetic complexes, representing key features distinguishing them from cultivated methanogenic relatives. Remarkably, back flux experiments revealed that the anaerobic oxidation pathways for volatile alkanes in certain archaeal lineages are fully reversible (68, 69, 111), capable of converting CO_2_ back to alkanes via reverse operation of the oxidative pathway. These findings point to the presence of archaea-mediated alkanogenesis in nature, which may facilitate the explanation of cryptic hydrocarbon production observed in sedimentary ecosystems (112–116).

## ECOLOGICAL NETWORKS INVOLVED IN HYDROCARBON BIODEGRADATION

Hydrocarbon-degrading microorganisms live as members of spatially structured, phylogenetically diverse, and metabolically interconnected communities (33). Complete oxidation of hydrocarbons is achieved by complex degradation networks involving a diversity of microbes with redundant or complementary metabolic activities (4). One type of network is implemented by multiple “single-performer” microorganisms endowed with all enzymes for hydrocarbon mineralization (27). In this scenario, the constituent taxa act in parallel and carry out complete biodegradation reactions independently. The presence of “single-performer” networks in natural environments is evidenced by SIP-metagenomics, which identified primary degraders encoding the complete genetic apparatus for degradation (117). Ecological interactions between multiple single performers in natural environments remain unclear. In principle, sharing the same growth substrates may lead to interspecies competition, whereas niche partitioning along other abiotic environmental variables may permit co-existence.

A second type of hydrocarbon degradation network is represented by microbial consortia defined by cooperative activities, with each member performing partial metabolic steps (Fig. 2). This scenario is formulated from the observation that full PAH degradation pathways were detected in none of the nearly complete MAGs from PAH-degrading communities in the Deepwater Horizon oil plume (10, 67). The missing steps of metabolic pathways are believed to be catalyzed by other community members with complementary functions. An essential element to such community-level cooperation is sharing a pool of freely diffusible metabolites, defined as epi-metabolomes, among constituent taxa—metabolic intermediates secreted by one member are captured and further metabolized by others (27). Indeed, metabolites such as acetate and hydrogen have been proposed as central intermediates maintaining the deep-sea microbial community nourished by hydrocarbons (79, 80). Another example of such interdependence is found in archaeal alkane oxidizers that lack pathways for terminal electron-acceptor reduction (118). Instead, these archaea rely on syntrophic interactions, transferring reducing equivalents extracellularly to partners, such as sulfate-reducing bacteria or methanogens (60, 69, 119). The epi-metabolomes allow cooperative activities of different species together, enabling a great number of reactions to occur at the community level. The main consequence is a maximization of hydrocarbon degradation. For example, a variety of species were shown to act cooperatively in mineralizing phenanthrene in soils (25), with *Sphingomonadales* dominating the early steps of degradation (e.g., initial hydroxylation) and *Actinomycetota* and *Bacillota* contributing more to downstream conversions (i.e., phthalate and protocatechuate pathway). Likewise, in phenanthrene-contaminated soils, degradation proceeds through a concerted action of diverse microorganisms, wherein unclassified *Deltaproteobacteria*, *Mycolicibacterium*, and *Mycobacterium* spp. mediate initial deoxygenation. The subsequent degradation steps, including fission of aromatic diols, cleavage of the secondary ring, and terminal conversion of phthalate, are carried out by distinct assemblages of soil microorganisms (120). These findings are consistent with the Black Queen theory, which assumes that interdependent cooperative interactions between bacterial species are established by complementary loss of shared diffusible functions, which in turn give a selective advantage to the microbial community as a whole (121, 122).

The functioning of such degradation networks seems to depend more on the presence of pools of essential species rather than on the total diversity of active hydrocarbon degraders. For example, phenanthrene degradation efficiency in soils relied on the relative abundance of a few phenanthrene-degrading taxa such as *Mycobacterium*, *Massilia*, and *Arthrobacter*, but was not positively correlated with species richness of active phenanthrene degraders (84). The data implied co-existence of both “driver” and “passenger,” referring to species with large or small effects on the ecosystem functioning by Walker’s hypothesis in hydrocarbon-degrading microbial guilds (123). Therefore, more than quantitative diversity (the number of distinct phylotypes), the qualitative diversity of degraders (the presence of “drivers”) might be more important in determining the efficiency of hydrocarbon degradation. Components other than microorganisms that degrade pollutants may also control the fluxes of degradation networks under certain conditions (124). For instance, within an anaerobic degradation system formed by fermenting microorganisms (degraders) and terminal electron-acceptor microorganisms (e.g., methanogens), mathematical modeling suggested that flux control may reside with terminal electron-accepting microorganisms under less favorable redox conditions (125). A better understanding of ecological networks involved in the degradation processes could guide the design of microbial consortia toward optimized performance. Ruan et al. (12) have developed a community-level metabolic model to simulate the performance of different microbiomes by accounting for the metabolic interactions between constituent species. Using this model, Ruan et al. (12) have assembled bioremediation-enhanced synthetic microbiomes based on keystone species identified from natural microbiomes (12).

Viruses, a previously overlooked ecological component, can also exert control on the composition and metabolism of microbial communities, having the potential to influence hydrocarbon degradation in nature (Fig. 2). In cold seep sediments, anaerobic gaseous alkane oxidizers, including *Methanomicrobia* and ANME, are widely infected by viruses (126, 127), suggesting hydrocarbon-degrading communities may be susceptible to viral infection and their population dynamics can be modulated by virus-mediated cell lysis. Moreover, viruses can directly reprogram the hydrocarbon degradation metabolism of the bacterial hosts through auxiliary metabolic genes (AMGs) expressed during infection. For instance, viruses of the family *Phycodnaviridae* in Arctic marine ecosystems were found to encode *almA* (flavin-binding monooxygenases for long-chain alkane degradation) and *ndoC* (naphthalene dioxygenase) (128), which may contribute to natural attenuation of hydrocarbons by alleviating metabolic bottleneck in host cells. Viruses encoding *badH*, one of key genes responsible for anaerobic benzoate/cyclohexanecarboxylate degradation, are also widespread across marine and terrestrial ecosystems (129). These suggest that viruses may have substantial direct or indirect contributions to global hydrocarbon degradation.

## CONCLUSIONS AND OUTLOOK

The coordinated application of SIP and metagenomics has extended hydrocarbon biodegradation studies from pure cultures to complex microbial communities, enabling a critical step toward uncovering the microorganisms, enzymes, and complex degradation networks that catalyze bioremediation processes *in situ*. Further progress will likely capitalize on deeper insights into genomic structure, physiological properties, and ecological behaviors of the key players that control the fate of hydrocarbons. Future advances in long-read sequencing technologies (i.e., Nanopore) combined with increasing sequencing depth will have great promise to improve the completeness of metagenome assemblies from environments. In turn, higher genomic resolution with more complete and contiguous MAGs could provide the foundation to develop constraint-based metabolic models capable of predicting microbial activities and metabolic interactions, including syntrophic relationships and metabolite exchange between species (105, 130, 131). Also, with the rapid progress in fluxomics (132) and metabolomics (133), in particular when combined with metagenomics, we anticipate a solid potential to untangle the intricate degradation networks that connect biodegradation reactions across members of microbial communities. Moreover, innovations in cultivation strategies are increasing our capabilities to bring the uncultured hydrocarbon-degrading microorganisms from environments to culture (62, 134, 135). Newly retrieved cultures offer the opportunity to investigate the physiological mechanisms underlying their biodegradation activities in contaminated environments (28). New frontiers in bioremediation are being opened by these fundamental insights into microbial degradation systems. Recent breakthroughs in synthetic biology and genome-editing techniques have resulted in an engineered strain that simultaneously degrades diverse organic pollutants, from monocyclic to polycyclic aromatic hydrocarbons, across diverse engineered environments (136). However, the environmental deployment of genetically engineered microorganisms could raise important biosafety concerns, including potential horizontal gene transfer to native microbial communities, ecological disruption through competitive displacement of indigenous populations, and unpredictable evolutionary trajectories in open systems. Therefore, rigorous risk assessment frameworks, containment strategies such as genetic biocontainment circuits, and comprehensive environmental monitoring are essential prerequisites before field-scale application of engineered strains in bioremediation practices. Future studies building on these advances will facilitate the development of increasingly sophisticated molecular tools to monitor biodegradation processes *in situ* and guide the implementation of targeted bioremediation strategies for improved efficacy.
